# Low risk of ocular toxicity following topical ocular administration of epinephrine nasal spray: implications for unintentional exposure

**DOI:** 10.1186/s13223-026-01040-2

**Published:** 2026-05-07

**Authors:** Anne K. Ellis, David M. Fleischer, Carlos A. Camargo, Richard Lowenthal, Sarina Tanimoto

**Affiliations:** 1 Division of Allergy and Immunology, Department of Medicine, Queen’s University, Kingston, ON Canada; 2 Section of Allergy and Immunology, Children’s Hospital Colorado, Aurora, CO USA; 3 Massachusetts General Hospital, Harvard Medical School, Boston, MA USA; 4 ARS Pharmaceuticals Operations, Inc., El Camino Real, Suite 300, San Diego, CA 11682 USA

**Keywords:** Anaphylaxis, Epinephrine, Intranasal epinephrine, Ocular, Toxicity

## Abstract

**Introduction:**

Unintentional ocular exposure to epinephrine nasal spray (10 mg/mL epinephrine) is a potential safety concern. Although epinephrine is used in multiple ocular formulations, unintentional ocular administration of epinephrine nasal spray was assessed to determine tolerability and support the overall safety profile.

**Methods:**

A non-GLP ocular tolerability study was conducted using six naïve New Zealand White rabbits (3 males, 3 females). Vehicle control or epinephrine nasal spray (1 mg) was applied to the right or left eye, respectively, once on Day 1. Animals were monitored over seven days for mortality, clinical signs, body weight changes, ophthalmic findings, ocular irritation (using the Modified Hackett-McDonald Scoring System), and gross necropsy findings.

**Results:**

Epinephrine nasal spray was well tolerated with no mortality, clinical abnormalities, or changes in body weight. Ophthalmic examinations via indirect ophthalmoscopy and slit-lamp biomicroscopy revealed no signs of irritation or ocular toxicity. No gross pathological changes were noted at necropsy.

**Conclusion:**

A single topical administration of epinephrine nasal spray (10 mg/mL) in rabbit eyes was not associated with any adverse effects, indicating a low risk of ocular toxicity. These results support the safety of epinephrine nasal spray in the event of unintentional ocular exposure and taken together with epinephrine’s use in other ocular formulations, suggest that no further nonclinical ocular studies are necessary.

Epinephrine nasal spray (neffy in the US or marketed as EurNeffy in certain regions) is a novel intranasal epinephrine product developed by ARS Pharmaceuticals and approved by the U.S. Food and Drug Administration for the emergency treatment of Type I allergic reactions, including anaphylaxis [1]. The introduction of a needle-free epinephrine delivery product represents a significant advancement in the treatment of severe allergic reactions, with the potential to improve treatment outcomes by removing barriers to prompt administration, particularly for individuals who are anxious about needles/injections.

One theoretical concern raised with all nasal sprays is the possibility of unintentional ocular exposure. Although epinephrine has a long history of ophthalmic use in formulations for glaucoma [2] and other indications [3, 4], the local tolerability of nasal epinephrine following unintentional ocular administration had not yet been characterized.

To address this gap, we conducted a non-good laboratory practice ocular tolerability study in six naïve New Zealand White rabbits (three males, three females). Rabbits are commonly employed in ocular toxicology assessments due to the relative size of the eye, ease of examination, and established sensitivity to irritants. Each animal received a single topical ocular dose on Day 1, with nasal epinephrine spray 1 mg administered to the left eye and placebo (nasal epinephrine spray formulation without epinephrine) administered to the right eye.

Animals were observed over a seven-day period with frequent in-life assessments, including mortality checks, general clinical signs, daily cage-side observations, and scheduled ophthalmic examinations performed with slit-lamp biomicroscopy. Body weight was measured at baseline and periodically thereafter. Ocular irritation was scored according to the Modified Hackett–McDonald scoring system, a standardized method that evaluates conjunctival redness, discharge, chemosis, corneal opacity, and iris involvement. Gross necropsy was performed at study termination to identify any macroscopic abnormalities.

No mortality or systemic clinical signs were recorded. Body weights remained stable throughout the observation period, with no evidence of treatment-related weight loss. Ocular irritation scores were 0 for all parameters throughout the study, indicating no evidence of conjunctival redness, discharge, swelling, corneal opacity, or iris involvement. Transient mydriasis was observed in the treated eye at 30 min and 1 h postdose and resolved by 6 h, consistent with the known pharmacologic effects of epinephrine. At necropsy, all animals had unremarkable ocular and systemic findings.

These results demonstrate that unintentional ocular exposure to nasal epinephrine spray was well tolerated in this rabbit model. Importantly, no clinically meaningful ocular irritation or toxicity was observed. The absence of ocular toxicity provides reassurance that unintentional ocular exposure to nasal epinephrine spray during real-world use is unlikely to result in any significant safety or tolerability concerns. This strengthened confidence in nasal epinephrine spray’s overall risk–benefit profile [5–9] for the emergency treatment of severe allergic reactions, including anaphylaxis.

## Data Availability

No datasets were generated or analysed during the current study.
